# Hastening Time to Ejaculation in Donkey Jacks Treated with the PGF2α Analog, Cloprostenol Sodium

**DOI:** 10.3390/ani10122231

**Published:** 2020-11-27

**Authors:** Duccio Panzani, Miguel Quaresma, Diana Fanelli, Francesco Camillo, Rebecca Moroni, Alessandra Rota, Ana Martins-Bessa, Miguel Nóvoa, Jaime Catalán, Igor F. Canisso, Giuseppe Conte, Jordi Mirò

**Affiliations:** 1 Department of Veterinary Clinical Sciences, University of Pisa, via Livornese 1286, San Piero a Grado, 56122 Pisa (PI), Italy; diana.equirepro@gmail.com (D.F.); francesco.camillo@unipi.it (F.C.); r.moroni1@studenti.unipi.it (R.M.); alessandra.rota@unipi.it (A.R.); 2 Center of Animal and Veterinary Science (CECAV), University of Trás-os-Montes e Alto Douro (UTAD), Quinta de Prados, 5000-801 Vila Real, Portugal; miguelq@utad.pt (M.Q.); abessa@utad.pt (A.M.-B.); 3 Association for the Study and Protection of Donkeys (AEPGA), Largo da Igreja, 5225-011 Atenor, Portugal; miguelnovoa@aepga.pt; 4 Equine Reproduction Service, Department of Animal Medicine and Surgery, Veterinary Faculty, Autonomous University of Barcelona, E-08193 Bellaterra, Cerdanyola del Valles, Spain; dr.jcatalan@gmail.com (J.C.); Jordi.Miro@uab.cat (J.M.); 5 Department of Veterinary Clinical Medicine, College of Veterinary Medicine, University of Illinois Urbana-Champaign, Champaign, IL 61802, USA; canisso@illinois.edu; 6 Department of Agriculture, Food and Environment, University of Pisa, via del Borghetto 80, 56124 Pisa, Italy; giuseppe.conte@unipi.it

**Keywords:** donkey, semen collection, cloprostenol sodium, erection, ejaculation

## Abstract

**Simple Summary:**

Semen collection in donkey jacks can last up to 90 min due to the long courtship needed for this species’ males to obtain sexual excitation and erection. In several domestic animals, ProstaglandinF2α successfully stimulated excitement in the male prior to collection and enhanced semen production. In our study, the prostaglandin analog cloprostenol sodium, administered prior to the semen collection, hastened erection and ejaculation in almost all donkey jacks. No differences have been found in semen production compared to control.

**Abstract:**

Due to the long courtship needed to attain excitation and erection, donkey semen collection can take up to 90 min. ProstaglandinF2α (PGF2α) has been reported to hasten the onset of erection and ejaculation in domesticated mammals, presumably by inducing smooth muscle contractions in the internal genitalia. However, while it has been anecdotally used in donkeys, it has yet to be critically evaluated. This study aimed to compare behavioral and semen parameters in Catalan, Balearic, Amiata, and Miranda jacks treated with the PGF2α analogue cloprostenol sodium immediately prior to exposure to an estrus jenny. Nineteen donkeys were assigned in a crossover design to receive cloprostenol sodium (125 µg, i.m.; *n* = 53 collections) or saline (1 mL, i.m.; *n* = 53 collections). There were no differences for erection (52/53 vs. 52/53) or ejaculation (52/53 vs. 48/53) for collection attempts assigned to saline or cloprostenol sodium, respectively. Cloprostenol sodium significantly hastened treatment-to-erection and treatment-to-ejaculation times from 12.0 ± 1.6 to 6.0 ± 1.6 min and from 14.0 ± 1.4 to 9.6 ± 1.4 min, respectively. Significant effects of breed and age were observed in behavioral and parameters, but there were no effects of cloprostenol sodium administration on semen parameters. In conclusion, cloprostenol sodium administration immediately prior to semen collection hastened time to collect semen in donkeys with no detrimental effects on semen quality and can be used by practitioners to circumvent long delays in donkey semen collection.

## 1. Introduction

Throughout the centuries, domestication of donkeys has had numerous functions, such as transportation, plowing plantation fields, mining, packing animals, and producing mules (hybrids with horses) [1,2,3]. After the mechanization of agriculture, donkeys lost their value as agricultural commodities in industrialized [4] and developing countries [5]. However, in developing countries, donkeys still playing a paramount role as a valuable agricultural commodity [5].

The decreased importance of donkeys and their hybrids, particularly in Western Europe, has resulted in a drastic reduction in donkey numbers. Most importantly, European donkey breeds are at risk of extinction or have a low minimal critical number (<500) of animals recommended by the conservation entities [4,6,7]. One of the ways to circumvent extinction is developing germplasm banks of cryopreserved semen and embryos [6]. However, donkeys present a particular challenge during intensive breeding management situations, as semen collection cannot, at times, be consistently carried out. For instance, weather conditions or minor changes in management can drastically affect time to collect semen, especially in young donkeys that take much longer to collect than older mature donkeys [8]. In the American continent, donkeys are used primarily to produce show mules or to herd beef cows [9]. As most mares are not receptive to donkeys [10], semen is typically collected, extended, and mares are artificially inseminated [6,9]. Under intensive regimen of semen collection for cryopreservation or immediate artificial insemination, procurement of semen in a consistent and reasonable timely fashion is highly desirable and challenging in donkeys [8]. Collecting semen from donkey jacks is often a time consuming and frustrating practice. The time needed to obtain an erection followed by ejaculation varies from 6 to 32 min when mounting estrus jennies [11,12,13] or estrus mares [14]. In a study of natural mating, the time between the introduction of the jack in the ‘jennies’ pen and the first mount with ejaculation varied between 25 and 93 min [15]. This study illustrated that donkeys could take a long time to collect semen and take a long time to naturally cover jennies. 

The luteolytic activity was the first reproductive action described for Prostaglandin F2α (PGF2α) [16]. Prostaglandin F2α is known to induce smooth muscle contractions in several species’ male internal genitalia, including humans, horses, and bulls [14,17]. This effect has been used to influence the volume and composition of the ejaculate in domesticated mammals [15,17,18,19,20,21,22]. Administration of PGF2α immediately before semen collection increases the total sperm number ejaculated in bulls, rabbits, and stallions [17,18,19,20,21].

Synthetic prostaglandins with more potent luteolytic activity have been developed in the last two decades. Uterine contractibility effect shows a high variability depending on the type of prostaglandin and the stage of the estrous cycle. Cloprostenol, a PGF2α analog, induces uterine contractions, nevertheless, causes no significant changes in any stage of the estrous cycle in cows [23]. Administration of PGF2α analogs immediately before semen collection enhanced libido of boars with known decreased sex drive [20,24] and reduced the time needed to train young boars for collecting semen and the number of false mounts in boars trained to the dummy mount [25]. Similarly, the administration of PGF2α to low libido bulls improved their libido [21]. It is unclear how PGF2α administration enhances sexual behavior in bulls or boars, but it is known that PGF2α can stimulate several areas of the brain in sows, which increase C-Fos mRNA expression and brooding behavior [26]. In horses, repeated treatment with PGF2α prior to semen collections initially increased the number of ejaculated sperm, and then an increase in semen volume and a reduction in sperm concentration was observed after 18 injections in nine weeks [27]. The same study reported no apparent deleterious effects on sperm production and quality associated with nine weeks and 18 injections of PGF2α.

Prostaglandin F2α administration has been used in clinical practice to circumvent problems with low libido in donkeys and to reduce time to collect semen under an intensive breeding management regimen [6,9]. However, the use of this eicosanoid has not been tested under controlled conditions. An increase of PGFM, an inactive metabolite assessed as a proxy of PGF2α, has been found in the plasma of jacks right before and after ejaculation time [13,17], suggesting that PGFα plays a role in erection and ejaculation in this species.

The objectives of this study were to assess semen and behavioral parameters of four European donkey breeds treated with cloprostenol sodium. We hypothesized that the administration of PGF2α pre-courtship reduces the time to collect semen in donkeys successfully.

## 2. Materials and Methods

This study was conducted after revision and authorization by the Research Ethics Committee of the Pisa University Ethics Committee under protocol# 8/2020, 31/01/2020.

### 2.1. Animals and Locations

A total of 19 jacks were enrolled in the study, and a total of 108 semen collections were attempted. The breeds of donkeys used in the study were as follows: Amiata (*n* = 4, age 6 ± 0 years, body weight 266 ± 26 kg, height: 135 ± 6 cm, Body Condition Score 3.1 ± 0.1), Balear (*n* = 4, age 8 ± 5 years, body weight 333 ± 20 kg, height: 142 ± 2 cm, Body Condition Score: 3.6 ± 0.5), Catalan (*n* = 5, age 6 ± 2 years, body weight: 406 ± 4 6 kg, height: 154 ± 4 cm, Body Condition Score: 3.5 ± 0.0), and Miranda (*n* = 6, age: 8 ± 6 years, weight 272 ± 34 kg, height 132 ± 5 cm, Body Condition Score 3.2 ± 0.4). The study was performed during the spring of 2020 in three research centers located in Pisa, Italy (Amiata), Barcelona, Spain (Catalan and Balearic), and Vila Real, Portugal (Miranda). Body condition score was calculated using the scale going from 1 to 4 described by Vall et al. [28].

During each semen collection, a jenny in good standing estrus was used as a mounting female for all three locations. Good standing estrus was defined as standing to be mounted and full display of “mouth clapping” [29]. One week before the beginning of the study, clean out semen collections were performed Monday, Wednesday, and Friday to standardize extra-current duct sperm reservoirs and to minimize possible effects of sexual inactivity. During the study, all jacks were housed in individual stalls far away from females to avoid audio and visual stimulatory effects. Jacks were collected with a Colorado (Amiata) or Hannover (Catalan, Balearic, and Miranda) artificial vagina models. After the cleanout week, jacks in each of the three research centers were randomly assigned in a cross over design between a treatment and control group. Immediately before exposure to an estrus jenny, the treatment group received cloprostenol sodium (125 µg cloprostenol sodium, i.m., 0.5 mL Estrumate^®^, MSD Animal Health SRL, Milan, Italy, PGF), whereas the control group received 1 mL of saline, i.m. (CTRL). After the cleanout week, jacks in each of the three research centers were randomly assigned in a crossover design, using the Latin square method. On consecutive collection days, the jacks would alternate between the treatment and control group. The order of collection between the different jacks would also be different in each collection session. Each Amiata, Catalan, and Miranda jack was submitted to three collections preceded by saline and three collections preceded by cloprostenol sodium treatment. Balearic donkeys instead had a total of two collections preceded by saline and two collections preceded by cloprostenol sodium treatment, following the same collection design used for the other breeds. Seventy-two hours of an interval between two collections were followed throughout the experiment across all breeds.

### 2.2. Behavioral Assessment

During all semen collection attempts, one observer recorded jacks’ sexual behavior in the presence of an estrus jenny. Specifically, the number of mounts, number of erections, time from treatment to erection, and time from treatment to ejaculation were recorded and used for comparisons across groups and breeds. The number of erection failures, ejaculation failures, persistent erections, and the number of sweating animals were accounted for and used for comparisons across groups and breeds. It was deemed erection failure if a jack did not achieve an erection by 90 min after exposure to an estrus female. Ejaculation failure was defined as the failure to successfully ejaculate within 100 min after exposure to an estrus jenny. Immediately after collection, jacks were assessed for a second persistent erection, every 15 min for 2 h after treatment administration. An erection lasting for 15 min or more was defined as “persistent erection”.

### 2.3. Semen Assessment

Immediately after collection, semen was assessed for total (pre-filtration) and gel-free (post-filtration) volume and concentration using a hemocytometer counting chamber. An aliquot of semen was extended (1:1) with a temperature-matched commercially available horse extender (INRA 96, IMV, l’Aigle, France). The extended semen was placed on a warm slide, covered with a coverslip, and then subjective sperm motility was assessed at 37 °C and 200× magnification under a phase-contrast microscope.

### 2.4. Statistical Analyses

Data analyses were carried out with JMP software (JMP 7 SAS Institute Inc., Cary, NC, USA). Fisher’s exact test was used to compare breeds, treatment regarding erection failure, ejaculation failure, persistent erection, and sweating after each collection attempt. A Shapiro–Wilk test was conducted to assess the normality of all variables and all dependent variables were shown to be normally distributed. Additionally, a linear model was created to determine the effects of multiple variables as described below.

_yijzpq_ = m + Breed_i_ + Treatment_j_ + Age_z_ + Treatment order_q_ + Breed_i_ × Treatment_j_ + Age_z_ × Treatment_j_ + Animal_k_(Breed_i_) + _eijzpq_

where:

_yijzpq_ = number of mounts, number of erections, treatment to erection interval (minutes), treatment to ejaculation interval (minutes), pre-filtration semen volume (mL), post-filtration semen volume (mL), subjective sperm motility (%), response sperm concentration (spz/mL), total sperm number.

m = mean

Fixed effects were age, breed, treatment, order of treatment. Jacks were accounted as random effect.

Breed_i_ = fixed effect of the _i_th breed (Amiata, Balearic, Catalan, Miranda).

Treatment_j_ = fixed effect of the _j_th treatment (CTRL, PGF).

Age_z_ = fixed effect of the _z_th age level (≤5, >5).

Treatment order_q_ = fixed effect of the qth treatment order level (6 levels).

Animal_k_ = random effect of the _j_th donkey (19 levels).

Since age is known to affect semen parameters and behavioral parameters [14], all jacks were grouped together and then split into groups according to age to assess those effects. Arbitrarily jacks were classified as young (i.e., ≤5 years old, *n* = 6), or mature (i.e., >5 years old, *n* = 13). Thereafter, data analyses were carried to assess the age and body weight effects on behavioral and semen parameters using the same similar linear model. Least-square means with their standard errors were reported, and treatment effects were declared significant at *P* < 0.05. The linear contrasts were tested in the first model by the *t*-test with Tukey’s adjustment within each parity level.

## 3. Results

An overall 3.7% erection failure was recorded in all 108 collections (Table 1). One Amiata donkey failed to achieve an erection while receiving saline treatment, and one Miranda jack failed to achieve an erection in the PGF group during a collection. Out of the 108 collection attempts, ejaculation failure was recorded in 5.5% of the collections (Table 1). One Amiata jack failed to ejaculate in one collection when assigned to the control group, whereas five jacks failed to ejaculate on one occasion (*n* = 1 Amiata, *n* = 1 Balearic, *n* = 2 Catalan, *n* = 1 Miranda) when assigned to the PGF group. There were no significant differences between groups regarding failures to achieve an erection or ejaculation (Table 1).

In all semen collection attempts, jacks performed at least one false mount. All jacks attaining an erection had it after interacting with the estrus jenny. In the PGF group, a second persistent erection, lasting for a minimum of 30 min, was observed after all collections in all the Amiata jacks and after one collection in a Miranda jack, respectively. Around thirty min of sweating was observed in two Miranda jacks in all cloprostenol sodium-treated collection attempts.

There were significant effects of breed, treatment, and breed by treatment interaction for behavioral parameters (Table 2). Cloprostenol sodium administration, compared to control, reduced the number of mounts per collection attempt (*P* = 0.025). Catalan and Balearic jacks had the lowest number of mounts per collection attempt in comparison with the other two breeds (*P* < 0.001) that were not significantly different from one another. The number of erections per attempt was affected by breed (*P* = 0.012), but not by treatment (*P* = 0.11), and it tended to have an interaction between breed and treatment (*P* = 0.06). There were effects of the breed (*P* < 0.001), treatment (*P* < 0.001), and breed by treatment interaction (*P* < 0.001) for the variables time from treatment to erection and time from treatment to ejaculation (Table 2). There were breed effects (*P* < 0.001) but no effect of treatment (*P* > 0.6) or breed by treatment interaction (*P* > 0.4) for all semen parameter evaluation (Table 3).

The number of mounts on the female before ejaculation was significantly higher in young compared to mature jacks (*P* = 0.001) as well as semen volume and total sperm number were higher in mature jacks compared to young ones (*P* < 0.001) (Table 4 and Table 5). Semen motility was, instead, higher in young ones (*P* = 0.021). Total sperm ejaculated was not significantly higher in the mature group.

**Figure 1:**
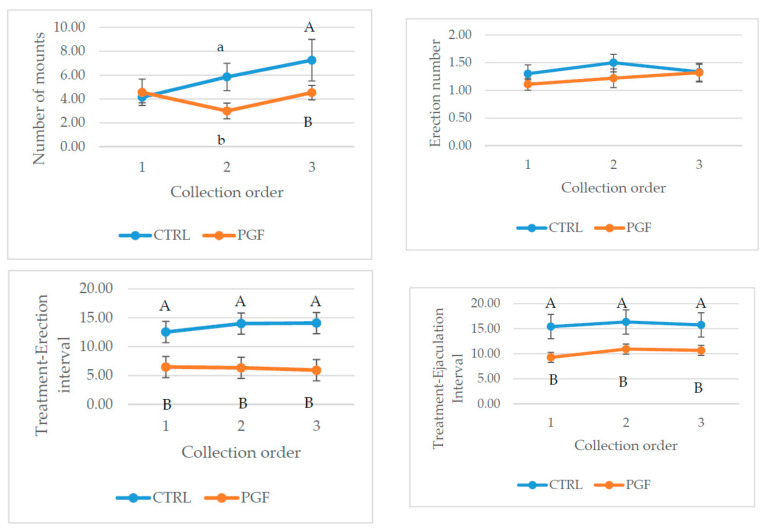
Effect of semen collection order (1, 2, or 3) and treatment (125 µg of cloprostenol = PGF, or 1 mL of saline = Control) interaction for behavioral features of donkeys: Jumps on teaser jenny, number of erections, treatment-erection interval (minutes), treatment-ejaculation interval (minutes). Data are expressed as least squared mean ± standard error. a,b: means ± SEM within rows and columns within the same analyzed parameter, with different letters significantly differ (*P* ≤ 0.05); A,B: means ± SEM within a column with different letters significantly differ (*P* ≤ 0.01).

There were effects of semen collection order for the number of mounts: In the second and third collection, a reduced number of mounts per collection attempts was observed in the cloprostenol sodium treated jacks, while treatment to erection and to ejaculation remained significantly shorter in the cloprostenol sodium treated group compared to the CTRL (Figure 1). No differences between treatment groups and semen collection order were found in seminal analyzed parameters (*P* > 0.05).

## 4. Discussion

This is the first study comparing the sexual behavior and semen parameters of donkey jacks receiving cloprostenol sodium to hasten time to semen collection. While studying donkeys in multiple centers, we evaluated four donkey breeds: The Balearic donkey is considered a large donkey breed, as well as the Catalonian, which is one of the founding breeds of the American Mammoth Jack; the Miranda, a Portuguese donkey breed, which would be considered a small standard jack based on size and the dairy Italian breed Amiata, which could be viewed as a standard size jack.

While we did not fully assess courtship behavior, cloprostenol sodium did not seem to drastically affect normal behavior, other than shortening the pre-copulatory time and decreasing the number of mounts without an erection. Worth noting that mounts with or without a full erection are part of a normal donkey courtship behavior during mating [15] semen collection mounting jennies [11] or mares. In the present study, cloprostenol sodium reduced the number of mounts per collection, likely because of the time to obtain an ejaculate. The dose of cloprostenol sodium used herein was half of the typical amount used to induce luteolysis in mares and jennies. The minimal effective dose of cloprostenol sodium has not been studied in donkeys or horses. In clinical practice, cloprostenol sodium (125–250 µg) or dinoprost (2.5–5 mg) have been used as either half or full luteolytic dose. Herein, half of the dose usually administered to induce luteolysis in equids was able to hasten the erection and ejaculation more rapidly and efficiently (with fewer false mounts before ejaculation) than the control collections. 

Erection was obtained in all but three collections (one after saline and two after cloprostenol sodium administration) after female contact, showing the necessity of sexual stimulation to awake sexual activity, and, probably, an exclusive action of cloprostenol sodium on the penis erectility, as demonstrated in vitro in several species [18,30]. The interval from courtship to mount and ejaculation in the donkey species is longer than in horses [12,13,15,30]. The inter-male variation regarding the minimal time necessary for semen collection or mating has been well documented. Our findings are certainly encouraging to suggest that cloprostenol sodium administration can be an alternative to hasten the time necessary to attain a semen collection in donkeys.

Although it is impossible to recreate an identical environment during semen in three breeding centers across Europe, attempts were carried out to keep the conditions as similar as possible. The authors conducted extensive discussions to ensure standard conditions across centers. Therefore, breed differences observed herein for all the variables (i.e., the number of false mounts, number of erections before ejaculation, and the interval between treatment/female contact and ejaculation, in both PGF treated and untreated jacks) are likely real. 

Ejaculate volume (pre- and post-filtration) and semen concentration varied with breeds but not between cloprostenol sodium vs. control treatment within the same breed. Semen concentration was just affected by breed, while treatment, age and weight had no influence on the number of ejaculated spermatozoa. Interestingly, 10 mg of dinoprost (natural PGF2α) resulted in increased post-filtration volume and decreased sperm concentration in horses [27]. Breed differences in reproductive parameters of donkeys have already been anecdotally reported [9]. One of the limitations of the present study is that a small number of animals represented each breed, thus, it is possible that if a larger number of jacks were enrolled in the study, the differences in breeds could have been different.

Surprisingly, age did not affect the behavioral parameters evaluated, except for the number of mounts per collection. These results could be real or be due to the fact that the number of animals in each group (i.e., ≤5 years old young, *n* = 5 vs. >5 years old mature, *n* = 14) was skewed. Previously, it has been shown that younger donkeys took longer times to be collected than mature donkeys [5,14]. In the present study, mature donkeys had greater pre- and post-filtration ejaculate volume and lower total motility, regardless of treatment received. Mature donkeys had greater total sperm ejaculated compared to young donkeys. This could be because three of the donkeys in the young group were still developing and not reached the supposed peak in sperm production expected to happen around five years of age.

Differences among subsequent treatments are not evident within treated and untreated jacks for all the parameters studied (differently than from the horse stallion [27]). No negative or positive effects on studied parameters could be attributed to this dose of cloprostenol sodium in jackasses included in this study.

The sweating response found in two Miranda jacks, subsequent to cloprostenol sodium treatment, is consistent with what was previously reported in horses [19], probably due to the release of epinephrine from the adrenal medulla [31]. The jacks having this side effect were the smaller of the jacks included in the study of their breed, a consequence of the dose/weight, and of a breed-enhanced sensibility to cloprostenol sodium could be advocated.

Persistent erection in all the Amiata jacks treated with cloprostenol sodium could be due to the spastic contractions of the smooth muscle apparatus of the reproductive tract caused by PGF2α as seen in several species, such as humans [18], horses, and bulls [27,32]. The absence of this side effect in two out of four breeds could be due to the inherent breed variations, or body size and the only case in 1/4 breeds included could be due to the individual and breed differences already shown in this species [33].

## 5. Conclusions

In conclusion, the administration of cloprostenol sodium was able to hasten erection and ejaculation without affecting semen quality. The present multi-centric study certainly supports the previously anecdotal use of cloprostenol sodium in clinical practice, a multi-breed controlled design study described herein.

## Figures and Tables

**Table 1:** Erection failure, ejaculation failure, persistent erection, and sweating in jacks treated with 125 µg of cloprostenol sodium (PGF) or 1 mL of saline (CTRL) prior to semen collection mount in an estrus jenny. Three semen collections were performed in each of the assigned groups (PGF vs. Control) at 72 h intervals for Amiata, Catalan, and Miranda, whereas only two collections per group were performed in Balearic jacks at 72 h intervals.

	Amiata (*n* = 4)		Balear (*n* = 4)		Catalan (*n* = 5)		Miranda (*n* = 6)		Total (*n* = 19)	
	CTRL	PGF	CTRL	PGF	CTRL	PGF	CTRL	PGF	CTRL	PGF
Erection failure	1/12 (0%)	0/12 (0%)	0/8 (0%)	0/8 (0%)	0/15 (0%)	0/15 (0%)	0/18 (0%)	1/18 (5.5%)	1/53 (3.7%)	1/53 (3.7%)
Ejaculation failure	1/12 (8%)	1/12 (8%)	0/8 (0%)	1/8 (12.5%)	0/15 (0%)	2/15 (13.3%)	0/18 (0%)	1/18 (5.5%)	1/53 (0.2%)	5/53 (9.4%)
Persistent erection	0/12 (0%)	12/12 (100%)	0/8 (0%)	0/8 (0%)	0/15 (0%)	0/15 (0%)	0/18 (0%)	1/18 (5.5%)	0/53 (0%)	13/53 (24.5%)
Sweating	0/12 (0%)	0/12 (0%)	0/8 (0%)	0/8 (0%)	0/15 (0%)	0/15 (0%)	0/18 (0%)	6/18 (33.3%)	0/53 (0%)	6/53 (11.3%)

There were no differences between groups for any of the variables assessed. Only the Miranda jacks sweated in response to PGF.

**Table 2:** Behavioral parameters in 106 semen collections performed in 19 jacks of different breeds (Amiata, *n* = 4; Balearic *N* = 4; Catalan *n* = 5; Miranda *n* = 6). In half of the collections, jacks were treated with 125 µg of cloprostenol sodium (PGF) or 1 mL of saline (CTRL) prior to semen collection mounting an estrus jenny.

Breeds (n)	Number of Mounts Mean ± SEM		Number of Erections Mean ± SEM		Treatment to Erection Mean ± SEM (min)		Treatment to Ejaculation Mean ± SEM (min)	
	CTRL	PGF	CTRL	PGF	CTRL	PGF	CTRL	PGF
Amiata (n = 4)	6.8 ± 1.5 ^a^	6.8 ± 0.9 ^a^	1.3 ± 0.2	1.5 ± 0.3	18.7 ± 3.0 ^A^	6.3 ± 0.4 ^C^	21.6 ± 3.0 ^a^	15.6 ± 5.0 ^b^
Balearic (n = 4)	3.6 ± 0.3 ^b^	1.1 ± 0.1 ^c^	1.2 ± 0.2	1.0 ± 0.0	18.4 ± 1.7 ^A^	6.7 ± 0.8 ^C^	21.0 ± 1.3 ^a^	9.1 ± 0.8 ^c^
Catalan (n = 5)	3.5 ± 0.2 ^b^	1.6 ± 0.2 ^c^	1.7 ± 0.2	1.1 ± 0.1	14.3 ± 1.1 ^B^	6.4 ± 0.6 ^C^	17.7 ± 0.8 ^b^	9.9 ± 0.8 ^c^
Miranda (n = 6)	6.9 ± 1.5 ^a^	4.8 ± 1.0 ^b^	1.1 ± 0.1	1.1 ± 0.2	7.4 ± 0.6 ^C^	6.0 ± 0.4 ^C^	8.1 ± 0.5 ^c^	7.2 ± 1.3 ^c^
Mean (n = 19)	6.0 ± 0.8 ^a^	4.0 ± 0.8 ^b^	1.0 ± 0.2	1.1 ± 0.2	12.0 ± 1.6 ^B^	6.0 ± 1.6 ^C^	14.0 ± 1.4 ^b^	9.6 ± 1.4 ^c^

^a–c^: Means within rows and columns within the same analyzed parameter, with different letters, significantly differ (*P* ≤ 0.05); ^A–C^: Means within a column with different letters significantly differ (*P* ≤ 0.01).

**Table 3:** Seminal parameters in 106 semen collections performed in 19 jacks of different breeds (Amiata, *n* = 4; Balearic *N* = 4; Catalan *n* = 5; Miranda *n* = 6). In half of the collections, jacks were treated with 125 µg of cloprostenol sodium (PGF) or 1 mL of saline (CTRL) prior to semen collection mounting an estrus jenny (*P* > 0.05).

	Semen Volume (mL)				Total Motility Mean ± SEM (%)		Sperm Concentration Mean ± SEM (×106/mL)		Total Sperm Ejaculated Mean ± SEM (×109)	
	Pre-Filtration Mean ± SEM		Post-Filtration Mean ± SEM							
	CTRL	PGF	CTRL	PGF	CTRL	PGF	CTRL	PGF	CTRL	PGF
Amiata (n = 4)	33.8 ± 3.1	45.4 ± 8.6	31.7 ± 3.2	43.6 ± 8.6	70.0 ± 3.2	173.6 ± 4.6	425.5 ± 93.2	555.7 ± 138.9	11.3 ± 1.9	19.4 ± 4.4
Balearic (n = 4)	79.7 ± 7.5	69.7 ± 7.2	78.1 ± 7.3	69.1 ± 7.2	87.0 ± 1.5	88.3 ± 1.5	175.3 ± 15.3	198.3 ± 17.9	13.0 ± 0.9	13.1 ± 0.9
Catalan (n = 5)	68.0 ± 6.5	64.3 ± 7.4	67.1 ± 6.3	63.9 ± 7.6	88.2 ± 1.4	85.3 ± 1.9	237.8 ± 21.7	214.6 ± 22.1	14.8 ± 1.1	12.9 ± 1.7
Miranda (n = 6)	88.2 ± 12.7	81.3 ± 10.1	74.6 ± 11.0	71.8 ± 10.2	65.7 ± 6.1	68.9 ± 6.4	266.4 ± 39.4	227.4 ± 41.6	17.9 ± 3.5	15.63 ± 3.5
Mean (n = 19)	74.0 ± 7.5	70.0 ± 7.5	66.3 ± 7.0	64.5 ± 7.0	74 ± 3.1	76.0 ± 3.1	274.7 ± 42.4	279.3 ± 42.4	14.45 ± 1.85	15.3 ± 1.85

There were no differences between groups for any of the variables assessed.

**Table 4:** Behavioral parameters in 106 semen collections performed in 19 jacks classified as young (≤5 years old) and mature (>5 years old). In half of the collections, jacks were treated with 125 µg of cloprostenol sodium (PGF) or 1 mL of saline (CTRL) prior to semen collection mounting an estrus jenny.

Groups	Treatment	Young (*n* = 6; Mean ± SEM)	Mature (*n* = 13; Mean ± SEM)
Number of mounts	CTRL	8.1 ± 1.7 ^A,X^	4.8 ± 0.6 ^B,X^
	PGF	4.8 ± 1.2 ^A,Y^	3.6 ± 0.5 ^B,Y^
	Total	6.5 ± 1.1	4.1 ± 0.4
Number of erections	CTRL	1.4 ± 0.2	1.2 ± 0.1
	PGF	1.1 ± 0.1	1.2 ± 0.1
	Total	1.2 ± 0.1	1.2 ± 0.1
Treatment to erection (min)	CTRL	11.5 ± 1.2	12.4 ± 1.6
	PGF	6.4 ± 0.4	6.2 ± 0.3
	Total	8.9 ± 0.9	9.32 ± 1.0
Treatment to ejaculation (min)	CTRL	13.6 ± 1.3 ^X^	14.3 ± 1.7 ^X^
	PGF	10.1 ± 1.3 ^Y^	9.4 ± 2.0 ^Y^
	Total	11.8 ± 0.9	11.8 ± 1.3

^A,B:^ means within a line with different letters significantly differ (*P* ≤ 0.01). ^X,Y:^ For each parameter, means within a column with different letters significantly differ (*P* ≤ 0.01).

**Table 5:** Seminal parameters in 106 semen collections performed in 19 jacks classified as young (≤5 years old) and mature (>5 years old). In half of the collections, jacks were treated with 125 µg of cloprostenol sodium (PGF) or 1 mL of saline (CTRL) prior to semen collection mounting an estrus jenny.

Groups	Treatment	Young (*n* = 6; Mean ± SEM)	Mature (*n* = 13; Mean ± SEM)
Pre-filtration semen volume (mL)	CTRL	50.4 ± 5.4	84.1 ± 7.6
	PGF	50.3 ± 5.7	78.0 ± 6.3
	Total	50.3 ± 3.6 ^A^	81.0 ± 4.8 ^B^
Post-filtration semen volume (mL)	CTRL	44.8 ± 5.7	75.9 ± 6.6
	PGF	46.3 ± 5.9	72.7 ± 6.3
	Total	45.5 ± 3.8 ^A^	74.3 ± 4.4 ^B^
Total motility (%)	CTRL	81.3 ± 2.4	71.1 ± 3.8
	PGF	80.8 ± 2.3	74.1 ± 3.7
	Total	81.1 ± 1.7 ^A^	72.5 ± 2.5 ^B^
Sperm concentration (×106/mL)	CTRL	271.6 ± 19.5	276.0 ± 47.5
	PGF	243.3 ± 31.1	295.2 ± 62.0
	Total	257.5 ± 12.0	285.7 ± 36.8
Total sperm ejaculated (×109)	CTRL	10.7 ± 1.0	17.7 ± 1.9
	PGF	11.2 ± 1.1	17.3 ± 2.2
	Total	10.9 ± 0.7	17.5 ± 1.4

^A,B:^ means within a line with different letters significantly differ (*P* ≤ 0.01).
